# Recent Developments in Chemical Synthesis with Biocatalysts in Ionic Liquids

**DOI:** 10.3390/molecules200916788

**Published:** 2015-09-15

**Authors:** Mahesh K. Potdar, Geoffrey F. Kelso, Lachlan Schwarz, Chunfang Zhang, Milton T. W. Hearn

**Affiliations:** Centre for Green Chemistry, School of Chemistry, Monash University, Melbourne, Victoria 3800, Australia; E-Mails: mahesh.potdar@monash.edu (M.K.P.); geoff.kelso@monash.edu (G.F.K.); lachlan.schwarz@monash.edu (L.S.); chunfang.zhang@monash.edu (C.Z.)

**Keywords:** enzyme catalysis, ionic liquids, bioseparation, whole cell biocatalysis, process intensification

## Abstract

Over the past decade, a variety of ionic liquids have emerged as greener solvents for use in the chemical manufacturing industries. Their unique properties have attracted the interest of chemists worldwide to employ them as replacement for conventional solvents in a diverse range of chemical transformations including biotransformations. Biocatalysts are often regarded as green catalysts compared to conventional chemical catalysts in organic synthesis owing to their properties of low toxicity, biodegradability, excellent selectivity and good catalytic performance under mild reaction conditions. Similarly, a selected number of specific ionic liquids can be considered as greener solvents superior to organic solvents owing to their negligible vapor pressure, low flammability, low toxicity and ability to dissolve a wide range of organic and biological substances, including proteins. A combination of biocatalysts and ionic liquids thus appears to be a logical and promising opportunity for industrial use as an alternative to conventional organic chemistry processes employing organic solvents. This article provides an overview of recent developments in this field with special emphasis on the application of more sustainable enzyme-catalyzed reactions and separation processes employing ionic liquids, driven by advances in fundamental knowledge, process optimization and industrial deployment.

## 1. Introduction

As a consequence of rapid developments in many fields of synthetic organic chemistry, researchers from both academia and industry now have to pay much greater attention to the detrimental effects of chemicals and chemistry processes on the environment. Against the backdrop of climate change and increasing public concern about global sustainability, the increasing needs for environmental protection and sustainable development are forcing chemists to rethink the way in which chemistry is conducted, resulting in new synthetic strategies that are more atom efficient, achieve better mass intensification factors, generate less waste and utilize less hazardous conditions through the deployment of the twelve principles of Green Chemistry [1].

Various approaches can be practiced in green organic synthesis, including ways to enhance atom utilization, replace stoichiometric reagents with catalysts, use benign solvents or solvent-free processes, design and generate safer chemical products that are biodegradable, and reduce or totally eliminate waste products. A variety of next-generation solvents, including liquid polymers, fluorous solvents, supercritical fluids and ionic liquids have been explored as greener alternatives to those conventional organic solvents that exhibit undesirable flammable, explosive or toxic properties.

Ionic liquids, as the name suggests, are liquids comprised of ions. In practical terms, it is desirable for an ionic liquid to exist in the liquid state at temperatures below 100 °C and, preferentially, at ambient temperature. Although ionic liquids have been known for at least 100 years, their application in various chemistry processes has only been studied extensively over the last three decades [2]. Research into ionic liquids has ranged from their application as solvents in electrochemistry [3], organic and inorganic transformations [2,4], biotechnology processes [5], as solar cell electrolytes [6], and as supported phases in organic chemistry [7,8]. For organic synthesis with transition metal catalysis, ionic liquids were initially employed as biphasic systems [9]. Since then, their application has expanded to include the development of task-specific ionic liquids, wherein the constituent ions of the ionic liquid are designed to fulfill the requirements of a specific reaction [10,11] or alternatively a biotransformation [12,13]. In addition, substrates have been covalently attached to an ionic liquid prior to the reaction, after which the desired product is isolated and the ionic liquid recycled [14,15].

Ionic liquids have long been considered as green alternatives to organic solvents, although extensive debate and some controversy have surrounded their “so called” green properties. For example, although a specific ionic liquid may have negligible vapor pressure and low flammability, the anions and/or the cations, which comprise the ionic liquid, may individually, both, or when in combination, be toxic. Therefore, like any other solvent, all the potential detrimental properties, e.g., toxicity and low biodegradability, of a specific ionic liquid needs to be assessed before it can be regarded as a green alternative to conventional organic solvents. In addition to these considerations, the production and life-cycle of an ionic liquid also needs to be evaluated in order to fully determine its “greenness” compared to conventional organic solvents. A recent study by Deetlefs and Seddon on the “greenness” of various ionic liquids made by laboratory-scale synthetic methods found that there is considerable room for improvement in terms of E-factor, energy consumption and purification [16]. Hence, for future industrial processes it will be desirable from the outset to design the ionic liquid to be easily and cleanly synthesized, to have *inter alia* the requisite green properties of low vapor pressure, low flammability and low toxicity, and possess practical working attributes such as low viscosity and easy recyclability, by taking advantage of the enormous combinatorial and structural diversity of ionic liquids in terms of their anion/cation composition [17,18].

Enzymes are nature’s catalysts and have been employed for millennia in the manufacture of food and beverage products. With the advent of genetic engineering and advanced biotechnology processes, their use has greatly increased over several decades in industry for the synthesis of important pharmaceuticals, agrochemicals and fine chemicals [19,20]. Due to their excellent catalytic, stereo-selective and chemoselective properties, low toxicity, high biodegradability and efficiency under mild reaction conditions, enzymes are regarded as excellent green catalysts. Whilst some enzymes need to be associated with biological membranes to elicit function, many enzymes exist in nature in an aqueous environment. Many enzymes have the specific ability to perform well in organic solvents. For example, in his pioneering work, Klibanov showed that certain enzymes are stable and can catalyze reactions in various organic solvents like hexane, toluene, acetonitrile or tetrahydrofuran [21,22].

This versatile aspect of enzymes has prompted chemists to explore their utility in ionic liquids as the combination of biocatalysts and ionic liquids have the potential to provide a more sustainable approach for organic transformations to be carried out, particularly when the substrates have poor solubility in water. Interest in the use of ionic liquids dates back to 1984, when Magnuson *et al.* [23] investigated the effect of ethylammonium nitrate on the activity and stability of the enzyme alkaline phosphatase. Subsequently, considerable research effort has been committed, particularly since 2000, to evaluate the impact of the physicochemical properties and the design of ionic liquids suitable for the stabilization or activation of enzymes in several different formats, such as immobilization onto solid carriers, as sol-gel encapsulation, as solid phase complexes or following chemical modification [24,25,26]. It is widely appreciated that many ionic liquids exhibit considerable toxicity and poor biodegradability, which preclude their use in many cases as “green” solvents, whilst the effects of ionic liquids on the functional status and solubility of enzymes are difficult to accurately predict with the current state of knowledge.

Nevertheless, it is well known that the effect of ions from low to relatively high concentrations, e.g., in excess of 4 M for ammonium sulphate, on enzyme activity and stability usually follows the Hofmeister series, whereby strongly hydrated ions (called kosmotropes) increase the water structure and thus the conformational stability of proteins, whilst weakly hydrated ions (called chaotropes) generally operate in the opposite way, e.g., destabilize protein conformation. However, as a generalization, the behavior of the anions of more hydrophilic ionic liquids tends to correlate well with the Hofmeister series. For example, Yang [27] and Zhao [28] demonstrated that an ionic liquid composed of a chaotropic cation and a kosmotropic anion can favorably influence an enzyme to retain good activity and stability. When both the anion and cation are chaotropic, then loss of activity and stability tend to follow. The more hydrophilic ionic liquids that combine powerful hydrogen bonding capacity tend to act as water-mimicking liquids with the ability to dissolve an enzyme for monophasic catalysis while retaining a high level of enzyme activity [29,30].

**Figure 1:**
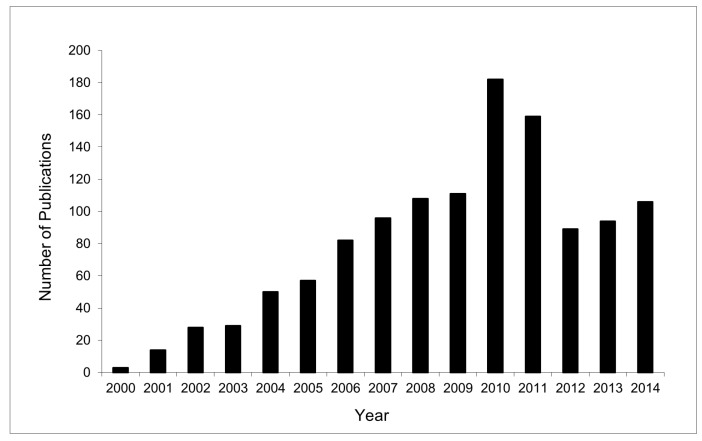
Year-wise reports published in the area of enzyme-based catalytic reactions in ionic liquids.

The first examples of the use of biocatalysts in the presence of an ionic liquid were reported by Erbeldinger *et al.* [31] and Lau *et al.* [32] in 2000. Since then, a large amount of research has been performed in this field as illustrated by Figure 1, which summarizes the number of publications per year from 2000. The number of issued or pending patent applications mirrors a similar trend. The importance of this rapidly growing field of research has been documented by various authors, including the different research groups of Sheldon *et al.*, Kragl *et al.* and Goto *et al.*, highlighting practical developments, as well as providing compilations of data for different ionic liquids/enzyme combinations [13,24,25,26,33,34,35,36,37,38,39,40,41]. Collectively, these works have summarized different facets of the use of biocatalysts in ionic liquids, wherein key issues related to the effects of ionic liquids on the structure, activity and stability of enzymes have been explored and the impact of the water content in the ionic liquid assessed, together with several biocatalytic reactions employing a range of different enzymes and ionic liquid combinations for the design of reaction systems, including biocatalyst recovery, product isolation and choice of biphasic systems.

In this current article, an overview of innovations that have been developed in this field over the last decade is presented with a focus on the synthetic chemistry outcomes and benefits derived from carry out biocatalytic reactions in suitable ionic liquids. Key aspects behind different chemical synthetic processes that are destined upon scale-up to truly be more sustainable are described. Emphasis has thus been mainly placed on the synthetic processes, including approaches whereby the product can be efficiently separated and the ionic liquid and biocatalyst can be recycled or employed in continuous processes, rather than on the physicochemical properties of the ionic liquids *per se*, which have been eloquently summarized in earlier reviews, (e.g., [2,5,13,16,24,25,26,33,34,35,36,37,38,39,40]). Importantly, such uses of biocatalysts in appropriate ionic liquids offers important advantages for process intensification and enhancement of synthetic productivity for the manufacture of chiral molecules. An overview of the application of a special class of biodegradable ion-based liquids, known as deep eutectic solvents, in biocatalysis, and recent adaptations of the use of ionic liquids to whole-cell biocatalysis, which from a green chemical perspective have potential for new applications in chemical manufacturing, medicine and the environmental sciences are also provided. 

## 2. Chemical Synthesis with Enzymes in Ionic Liquids

### 2.1. Ionic Liquids as Solvents for Enzyme Catalysis

**Scheme 1:**
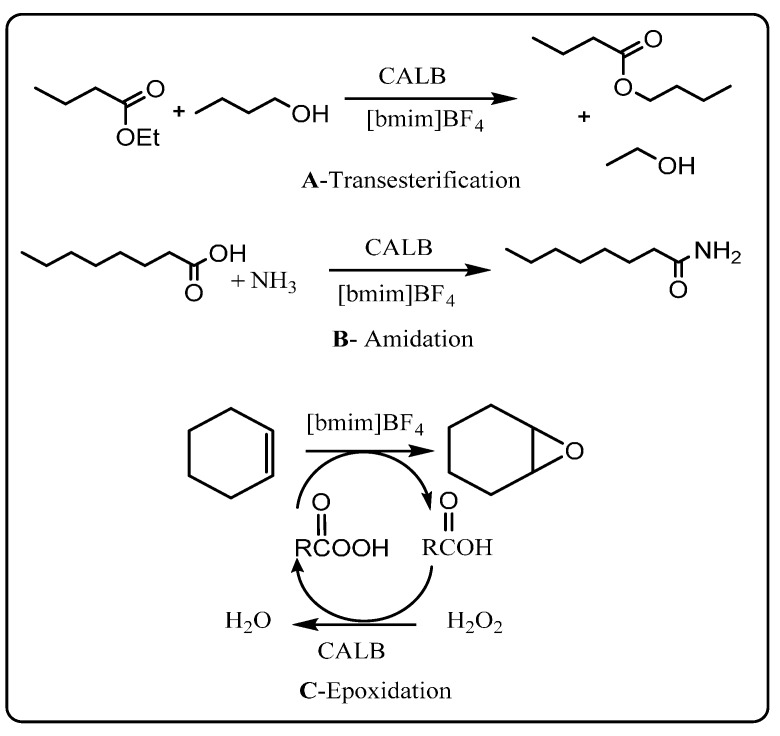
*Candida antarctica* lipase (CALB)-catalyzed transesterification (**A**); amidation (**B**) and epoxidation (**C**) reactions in ionic liquids [32].

The first definitive example of enzyme-catalyzed reactions in a pure ionic liquid was reported by Sheldon’s group. Thus, Lau *et al.* [32] reported lipase-catalyzed transesterification, amidation and epoxidation reactions in ionic liquids (Scheme 1). Reaction rates for the immobilized *Candida antarctica* lipase B (Novozym^®^ 435)-catalyzed transesterification in anhydrous [bmim][PF_6_] were found to be comparable, faster or slower than those in organic solvents depending on the type of ester and alcohol employed, with good to excellent conversions (56%–81%) obtained. The Novozym^®^ 435-catalysed condensation of ammonia with octanoic acid in [bmim][BF_4_] proceeded in quantitative conversion after 4 days. Epoxidation of cyclohexene by peroctanoic acid was also investigated using the Novozym^®^ 435 to catalyze the production of the peroxy-acid *in situ* from octanoic acid and 60% aqueous H_2_O_2_ in [bmim][BF_4_], documenting an example of a safer reaction process that did not require the use of a flammable solvent or the handling of a hazardous peroxy-acid. A yield of 83% was achieved after 24 h, comparable to that obtained when the same reaction was carried out in acetonitrile (93%). Overall, this pioneering study highlighted the potential of low vapor pressure, non-flammable ionic liquids as greener solvent alternatives to organic solvents for enzyme-catalyzed production of important industrial chemicals.

**Scheme 2:**
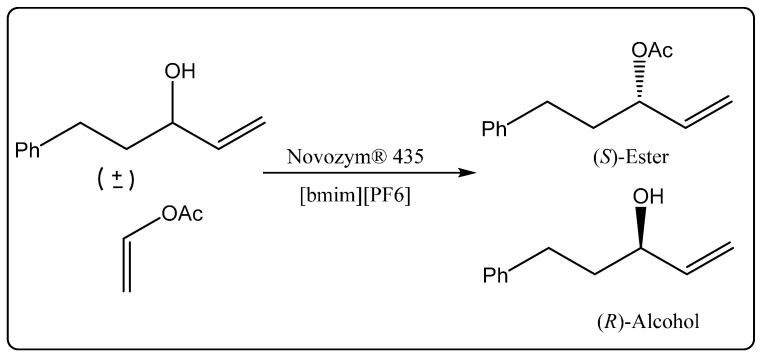
*Candida antarctica* lipase (CALB)-catalyzed enantioselective transesterification of allylic alcohols in ionic liquid [41].

The important roles that enzymes can play as catalysts are particularly prominent in enantioselective transformations. Pioneering examples of enantioselective reactions using enzymes in ionic liquids were demonstrated independently in 2001 by Schöfer *et al.* [40] and Itoh *et al.* [41]. For example, Itoh *et al.* [41] carried out the enantioselective acylation of allylic alcohols in an ionic liquid solvent. The group successfully demonstrated that the lipase *Candida antarctica* lipase B (Novozym^®^ 435) was anchored to the imidazolium based ionic liquid solvent. These experiments documented the power of a recyclable system of enzyme and ionic liquid for enantioselective transformation (Scheme 2). Overall, these demonstrations of the potential of lipase-catalyzed reactions in ionic liquids [32,40,41] represent landmark studies, since lipases are now the most widely used enzymes in organic synthesis.

**Scheme 3:**
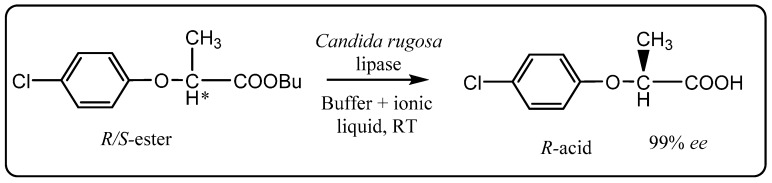
Enantioselective hydrolysis of racemic butyl-2-(4-chlorophenoxy)propionate in an aq. buffer/ionic liquid system [44].

Subsequent to this pioneering study of lipase-catalyzed biocatalysis in ionic liquids, several other different reactions encompassing a wider array of substrates have been reported [13,42,43]. For example, Mohile *et al.* have demonstrated the use of ionic liquid as a co-solvent with aqueous buffer for the *Candida rugosa* lipase*-*catalyzed enantioselective hydrolysis of racemic butyl 2-(4-chlorophenoxy)propionate (Scheme 3) [44].

**Scheme 4:**
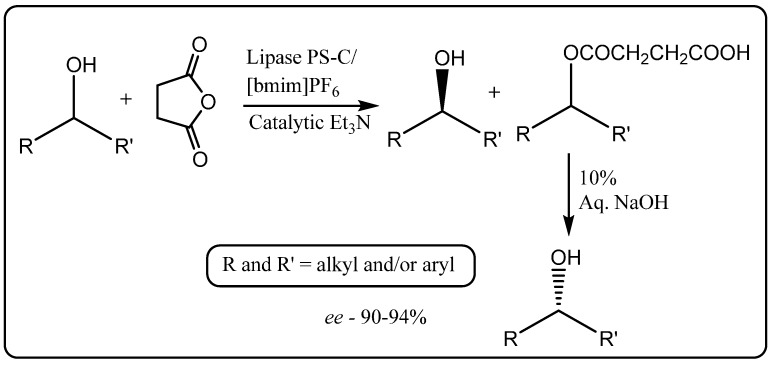
Lipase PS-C catalyzed resolution of racemic alcohols by enantioselective acylation with succinic anhydride in [bmim][PF_6_] [45].

In biphasic systems employing the hydrophobic [bmim][PF_6_] or 1-hexyl-3-methyl-imidazolium [hmim][BF_4_] ionic liquids, reaction times increased by about 10-fold compared to when an aqueous buffer alone was employed, but a remarkably large enhancement in enantioselectivity up to 99% compared to 47%, respectively, was observed. Similar results have been obtained with a monophasic system when the more hydrophilic [bmim][BF_4_] ionic liquid was used. In the case of the biphasic systems, the ionic liquids were able to be recycled with only marginally reduced conversion rates after four cycles and with no loss of enantioselectivity. In subsequent work [bmim][PF_6_] was assessed as a solvent for the resolution of racemic alcohols by enantioselective acylation with succinic anhydride using *Pseudomonas cepacia* lipase supported on celite (lipase PS-C) as a catalyst (Scheme 4) [45].

A distinct advantage of using succinic anhydride as the acylating agent lies in the easy separation of the resultant ester by extraction from the ionic liquid into a mildly basic aqueous solution, followed by hydrolysis with a strong base to give back the enantiomerically enriched alcohol. The ionic liquid [bmim][PF_6_] was found to be a suitable recyclable solvent for lipase PS-C-catalyzed enantioselective acylation, in up to 94% enantioselectivity. Including a catalytic amount of triethylamine as an additive enhanced the rate of the reaction by about 1.5-fold.

**Scheme 5:**
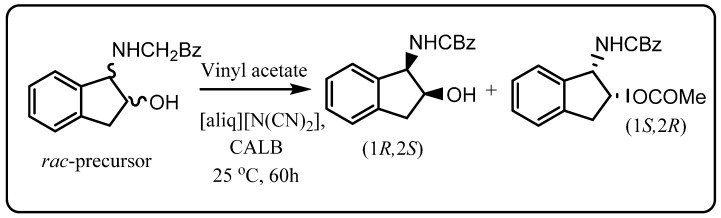
Resolution of *cis*-benzyl *N*-(1-hydroxyindan-2-yl)carbamate using CALB (Novozym 435^®^) in ionic liquid [46].

As an example of pharmaceutical synthesis, Lourenco *et al.* [46] developed an efficient lipase-mediated resolution of the HIV protease inhibitor Indinavir precursor (±)-*cis*-benzyl *N*-(1-hydroxyindan-2-yl) carbamate in [aliq][NCN_2_] ionic liquid (Scheme 5). Novozym^®^ 435 was again used to selectively acylate the 1*S*,2*R*-*cis*-benzyl enantiomer with vinyl acetate in up to 97% enantioselectivity. The catalyst was removed by filtration and the product and unreacted 1*R*,2*S*-enantiomer were then isolated from the non-volatile ionic liquid by sublimation, and then separated by column chromatography. This approach allowed reuse of the [aliq][NCN_2_] and enzyme with comparable yield and enantioselectivity.

**Scheme 6:**
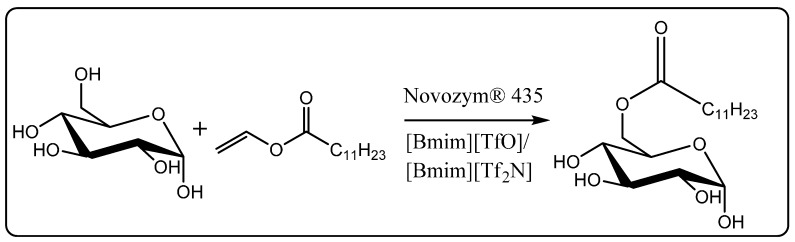
Transesterification of vinyl laurate with glucose in ionic liquids catalyzed by Novozym^®^ 435 [47].

Mai *et al.* have demonstrated the enzymatic synthesis of sugar fatty acids in ionic liquids [47]. Sugar fatty acid esters are environment friendly bio-surfactants known for their non-toxic, non-ionic, and high biodegradability. Transesterification of vinyl laurate with glucose was catalyzed by Novozym^®^ 435 in a mixture of [Bmim][TfO]/[Bmim][Tf_2_N] ionic liquid (1:1 *v*/*v*) in 96% conversion (Scheme 6). The mixture of [Bmim][TfO]/[Bmim][Tf_2_N] permitted good solubility of glucose (12 g/L) and enzyme stability critical for its reuse. The reaction was scaled up to 2.5 L with comparable conversion efficiency. After 10 cycles, the activity of Novozym^®^ 435 was 75% of the initial activity.

### 2.2. Developments in Sustainable Biocatalysis Employing Ionic Liquids

#### 2.2.1. Ionic Liquid-Coated Enzymes as Heterogeneous Catalysts

Initial applications of ionic liquids in biocatalysis concentrated on their use as a replacement for conventional solvents, with subsequent studies yielding greater understanding of how they interact with enzymes. An example of such an application was reported by Lee *et al.* [48], in which an ionic liquid-coated enzyme (ILCE) was prepared as a heterogeneous catalyst for use in the enantioselective transesterification of vinyl acetate with racemic alcohols in toluene. In this work, a lipase from *Pseudomonas cepacia* (PS) was first dissolved in the ionic liquid 1-(3′-phenylpropyl)-3-methylimid-azolium hexafluorophosphate [ppmim][PF_6_], which has a melting point of 53 °C. Upon cooling the solution to room temperature, a solid ILCE formed, which was then used as a heterogeneous catalyst for the conversion of the substrate to product in toluene. Compared to the native enzyme in toluene, the ILCE enhanced the enantioselectivity of the products by 1.5–2 folds. Furthermore, the ILCE was easily removed from the reaction mixture by filtration and reused as an ionic liquid coated biocatalyst for up to 5 times with comparable activity.

**Scheme 7:**
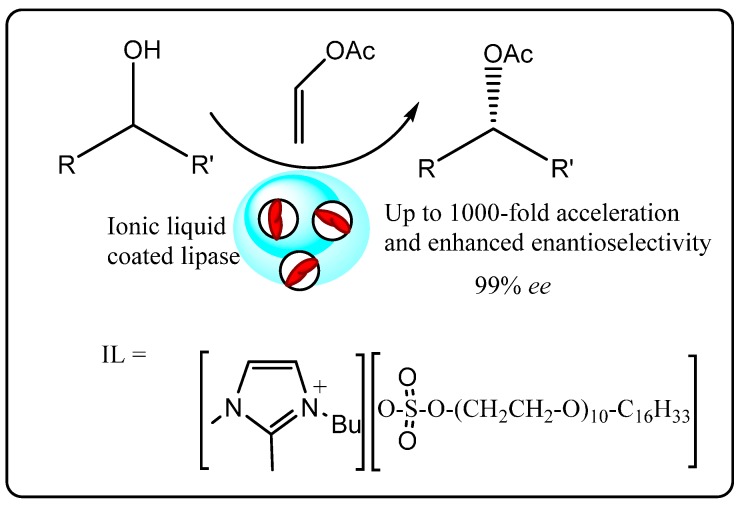
ILCE-catalyzed enantioselective transesterification of vinyl acetate with racemic alcohols [48].

Similarly, Itoh *et al.* [49] have prepared a 1-butyl-2,3-dimethylimidazolium [bdmim][cetyl-PEG10-sulfate] coated lipase by extracting a commercial lipase immobilized onto a ceramic matrix (lipase PS-C) into aqueous buffer containing the ionic liquid followed by freeze-drying. The ILCE was used as a heterogeneous catalyst for transesterification of vinyl acetate with racemic alcohols in diisopropyl ether (Scheme 7) and gave rate enhancements of up to 1000-fold compared to lipase PS-C while maintaining excellent enantioselectivity (>99%). Reusing the ILCE up to five times gave comparable product yield and enantioselectivity. To demonstrate the versatility of the protocol, similar activation effects were also demonstrated for [bdmim][cetyl-PEG10-sulfate]-coated *Candida Rugosa* lipase.

Electron microscopy of the ILCE showed it possessed a higher surface area than the commercial lipase PS-C immobilized on ceramic, whilst MALDI-TOF mass spectrometry experiments indicated the enzyme in the ILCE forms a discrete non-covalent complex with the ionic liquids. Together, these results suggested that the enhanced activity of the ILCE may be due to easier access of the substrate to the more porous ILCE and favorable enzyme flexibility and conformational changes imparted by its non-covalent interactions with the ionic liquid.

**Figure 2:**
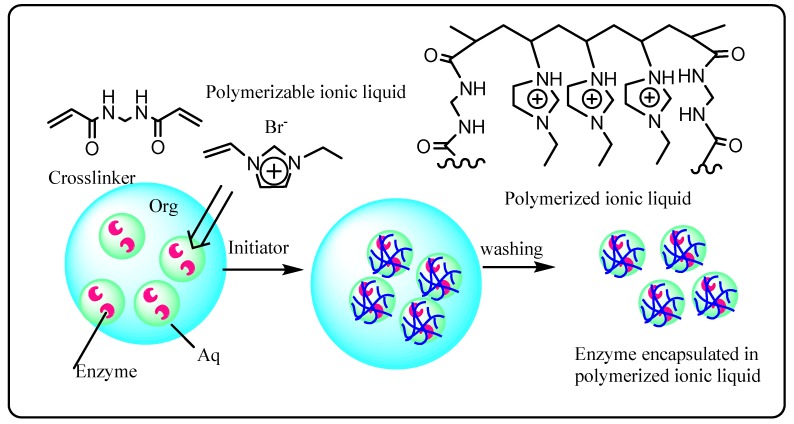
Enzyme encapsulation in an ionic liquid-based polymer [50].

Encapsulation of enzymes in polymers prepared from ionic liquid monomers has also been investigated [50]. A useful feature in designing ionic liquids is that pendant functional groups can be incorporated into the cation or anion for polymerization. Thus, 1-vinyl-3-ethylimidazolium bromide was polymerized with the cross-linker *N*,*N*′-methylenebis(acrylamide) in the presence of native horse-radish peroxidase (HRP) or HRP modified with a polyethylene comb polymer (PM_13_-HRP) to protect it from conformational deactivation by the ionic liquid bromide anion (Figure 2). This method led to the formation of enzyme-encapsulated polymer micro particles, which functioned as heterogeneous catalysts for the oxidation of guaiacol by H_2_O_2_ in water. The activity of the encapsulated PM_13_-HRP was found to be 3 times higher than that of encapsulated native HRP and 2 times higher than PM_13_-HRP encapsulated in non-ionic polyacrylamide micro particles. Furthermore, the encapsulated PM_13_-HRP was easily recycled by centrifugation, with reduction in activity occurring only at the first recycling stage, which was attributed to loss of PM_13_-HRP loosely immobilized on the surface of the micro particles.

**Scheme 8:**
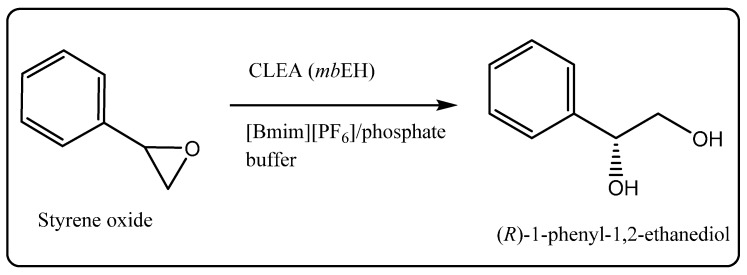
Cross linked enzyme aggregate of *mb*EH catalyzed asymmetric hydrolysis of styrene oxide [51].

Cross-linked enzyme aggregates (CLEAs) of mung bean epoxide hydrolases (*mb*EH) has been used to catalyze the asymmetric hydrolysis of styrene oxide in a water-ionic liquid biphasic system (Scheme 8) [51]. Styrene oxide remains dispersed in the ionic liquid [bmim][PF_6_] and the hydrolysis product, (*R*)-phenylethanediol ((*R*)-PED), was obtained in the aqueous phase, facilitating its easy separation from starting material and avoiding non-enzymatic hydrolysis. On a 500 mL preparative scale the yield of (*R*)-PED was 49% in 94.6% enantioselectivity.

Rehmann and co-workers have recently demonstrated that a variety of ionic liquids can stabilize the activity of the laccase from *Trametes versicolour* [52]. Laccase, an oxidative mediator system, is important owing to its potential for numerous green oxidation processes but its activity is inhibited by oxidation mediators like TEMPO (2,2,6,6-tetramethyl-1-piperidinyl)oxidanyl, ABTS (2,2′-azino-bis-(3-ethylbenzo-thiazoline-6-sulfonic acid) or 4-hydroxybenzyl alcohol. Such inactivation was avoided by using a biphasic ionic liquid-water system, wherein the mediator predominantly partitions into the ionic liquid, restricting its contact with the laccase, which predominantly resided in the aqueous phase.

#### 2.2.2. Role of Enzymes and Ionic Liquid System in Biomass Conversion and Biofuel Production

To address the expected increase in demand for energy and transport fuel from a rapidly increasing global population and developing economies, the sustainable conversion of renewable resources to liquid transport fuel and commodity chemicals has become an important area of research. Lignocellulosic (woody) biomass is the key sustainable biomass resource for transformation into biofuels and bio-based chemical products. However, the chemical composition of lignocellulosic biomass differs significantly from that of fossil fuels and efficient catalytic processes need to be developed in order to convert it to fuel precursors and platform chemicals, and the use of ionic liquids and enzymes in addressing this challenge is an active area of research.

Over the past decade, there has been increased interest in using cellulose from renewable biomass resources as a more sustainable alternative raw material to petrochemical feedstock for producing chemical products. A barrier to chemically transform cellulose to other products is its poor solubility in many different solvents. Although ionic liquids containing anions, which are strong hydrogen-bond acceptors, e.g., halides, have been found to dissolve cellulose, these types of ionic liquids tend to denature enzymes, hampering biocatalytic processing of the dissolved cellulose [53,54]. The recent observations of Zhao *et al.* [55] with polyethylene glycol-based ionic liquids that are able to dissolve carbohydrates and cellulose, and retain enzyme activity has provided one option to overcome these limitations. Moreover, these systems are also relevant to the transesterification of methylmethacrylate with glucose or cellulose using Novozym^®^ 435 with the biocatalytic reaction proceeded in up to 89% conversion and 66% yield [55].

**Figure 3:**
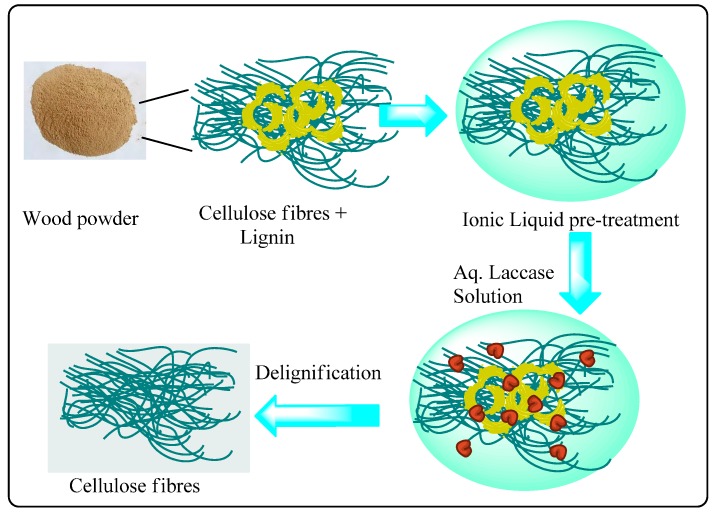
Delignification of woody biomass using ionic liquid and enzymes [60].

Currently, research efforts are also being focused on the use of ionic liquid and deep eutectic solvents for lignocellulosic pre-treatment [56,57] in conjunction with enzymatic hydrolysis of cellulose to fermentable sugars [58,59]. Lignocellulosic biomass is not amenable to facile microbial or enzymatic industrial biotransformation, which limits its economic conversion to fuel precursors and platform chemicals by a biotechnological process. Moniruzzaman and Ono have investigated ways to overcome this barrier by developing an enzymatic biomass process employing an ionic liquid pre-treatment to enable efficient access for enzyme laccase to reactive sites [60]. This enabled an efficient delignification in an ionic liquid/aqueous buffer system, giving cellulose fibers in improved yields and shorter reaction times (Figure 3).

**Figure 4:**
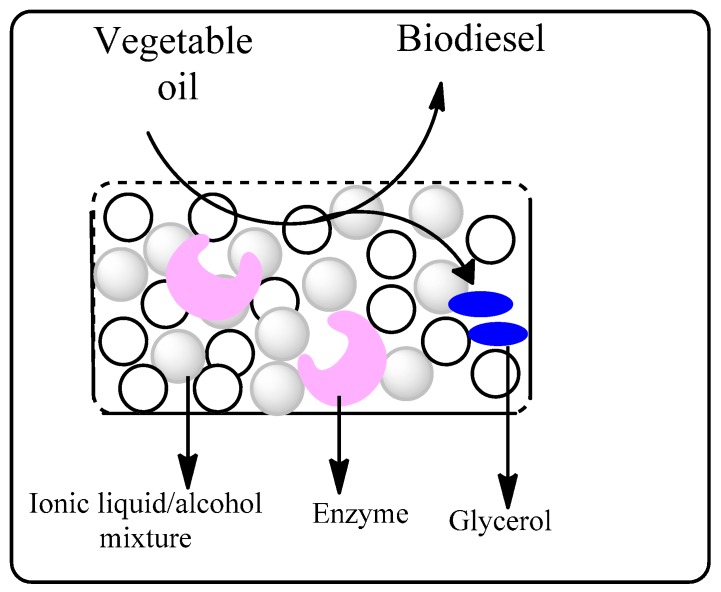
Lipase-catalyzed production of biodiesel in a biphasic ionic liquid-soybean oil system with concomitant separation of the glycerol by-product [63].

Biodiesel is a transport fuel that consists largely of fatty acid methyl esters (FAMEs) prepared by transesterification of natural triglycerides in plant oils or animal fats with methanol. These fuels have generated interest as renewable, low-carbon impact alternatives to fossil fuels. Current biodiesel production uses homogenous alkaline catalysts for transesterification and although this process is efficient, it complicates downstream product separation and increases energy costs [61]. Biocatalytic production of biodiesel in ionic liquids merits consideration as a green and potentially environmentally friendlier alternative to eliminate issues associated with product separation, homogenous catalyst recycling and energy consumption. The first demonstration of the potential of biocatalytic biodiesel production in ionic liquids was reported by Ha *et al.* [62], who used the Novozym^®^ 435 lipase to catalyze the transesterification of soya bean oil with methanol. 1-Ethyl-3-methylimidazolium trifluoromethanesulfonate ([Emim][TfO]) was found to be the best solvent, giving an 80% yield of fatty acid methyl esters in 12 h compared to 65% in *tert*-butanol and 10% under solvent-free reaction conditions. An additional advantage of the ionic liquid system was phase separation of the biodiesel as it was formed. Gamba *et al.* subsequently developed an efficient biphasic system for biodiesel production comprised of a [bmim]bis(trifluoro-methylsulfonyl)-imide ([NTf_2_]) ionic liquid phase containing methanol and lipase, and a soya bean oil phase (Figure 4) [63].

This transesterification proceeded at room temperature to give biodiesel in 95% yield in 24 h and was enhanced by the addition of water. Furthermore, the ionic liquid phase served as an extracting agent for removing glycerol from the reaction mixture and the biodiesel was separated by simple decantation. The ionic liquid phase containing the enzyme was recycled four times without significant loss of catalytic activity. After these four cycles the enzyme was filtered from the ionic liquid and the glycerol by-product extracted with water in 98% purity.

**Figure 5:**
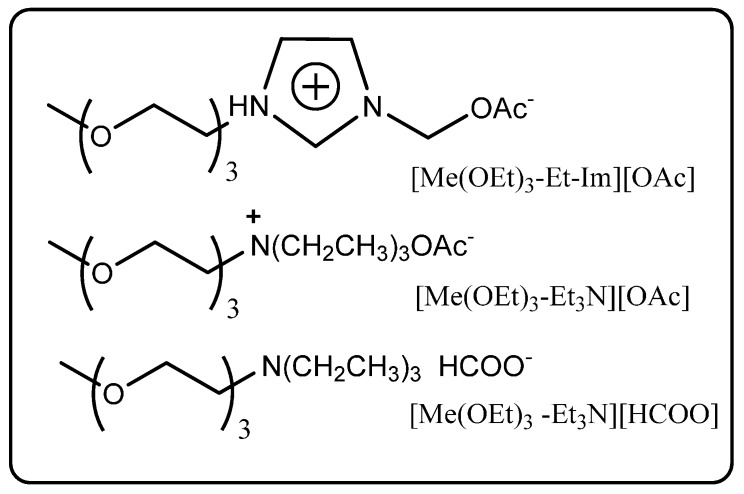
Novel triethyleneglycol-functionalized ionic liquids for biodiesel synthesis [64].

Further, Zhao *et al.* have reported the synthesis and application of novel triethyleneglycol-functionalised ionic liquids (Figure 5) in biodiesel production [64]. These ionic liquids dissolved both triglycerides and lipases, and a high level of catalytic activity was observed with different commercially available lipases; quantitative conversions of Miglyol^®^ oil 812 prepared from coconut and palm kernel oils was achieved at 96 h in a 70/30 (*v*/*v*) ionic liquid/MeOH mixture. Overall, these preliminary studies on the transesterification of vegetable oils in ionic liquids indicate they are promising solvent systems for industrial biocatalytic biodiesel production.

The enzyme catalysed esterification of soybean oil in [Emim][PF_6_] with microwave heating has been demonstrated by Yu *et al.* to give faster conversion with a 1.8-7.8-fold increase in enzyme activity compared to *t-*butanol and a solvent-free system using conventional heating [65]. This is the first example of using synergistic effect of microwave irradiation and ionic liquids for enzyme catalyzed biofuel production. The study also demonstrated recycling of the ionic liquid/enzyme phase for five cycles with only a slight decrease (8%) in enzyme activity.

Zhang *et al.* have demonstrated the activity of *Penicillium expansum* lipase (PEL) in ionic liquids and reported the application of this system for the synthesis of corn oil-based biodiesel [66]. The ionic liquid [Bmim][PF_6_] was found to be an optimal solvent for the transesterification reaction, giving a higher conversion (86%) compared to *t*-butanol (52%), and PEL was found to be more tolerant to the reaction conditions compared to other lipase enzymes routinely used. Furthermore, the biphasic ionic liquid-FAME reaction system facilitated separation of the biodiesel and recycling of the catalyst. Lai *et al.*, from the same group, have reported the use of PEL for the transesterification of triglycerides from the microalgae *Chlorella vulgaris*, the first example of ionic liquid mediated biocatalytic conversion of microalgal oil to biodiesel [67].

#### 2.2.3. Ionic Liquid-Based Supported Liquid Membranes in Separation Processes

Selectively permeable membranes that separate bulk solvent phases have potential application in separation processes. For example, kinetic resolution employing enzyme catalysis followed by product separation using selectively permeable membranes allows the conversion and separation of the desired product in a single continuous process which is attractive from an environmental and industrial perspective. Supported liquid membranes (SLMs) have been identified as suitable materials for such continuous separation processes. These materials are usually comprised of a solvent immobilized in the porous structure of polymeric or ceramic membrane [68]. The SLM serves to separate a feedstock solvent and a receiving solvent with the immobilized solvent mediating solute transport across the membrane. A critical feature of SLMs in continuous extraction processes is negligible loss of the immobilized solvent to either of the solvent phases and to the atmosphere.

The potential of using SLMs incorporating ionic liquids as the immobilized solvent for transport of organic molecules was first demonstrated by Branco *et al.*, who employed [bmim][PF_6_] in a polyvinyl-idene fluoride membrane owing to its poor solubility in the feedstock and receiving solvents, and negligible vapor pressure [69]. This system was able to selectively transport diisopropylamine from a mixture of amines in the feed solvent to the receiving solvent.

**Scheme 9:**
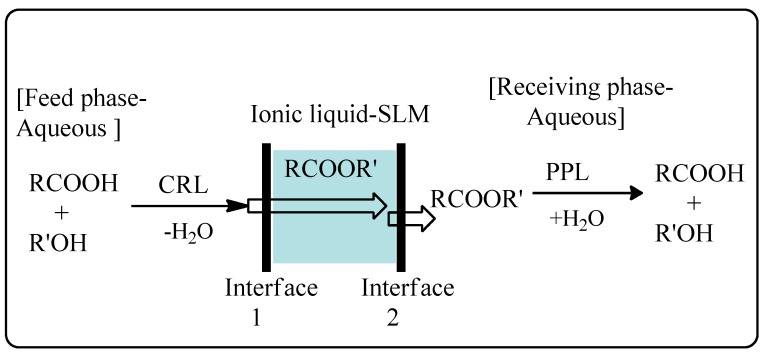
Lipase-facilitated transport of carboxylic acids across an ionic liquid-based supported liquid membrane (SLM) [70].

Miyako *et al.* have subsequently demonstrated a lipase-facilitated transport of organic acids across SLMs employing ionic liquids as transport solvents (Scheme 9) [70]. Based on this technology, organic acids were esterified by *Candida rugosa* lipase (CRL) in the feedstock phase with the resulting ester partitioning into the ionic liquid phase in the SLM and then diffusing into a receiving phase that contained a porcine pancreatic lipase (PPL) to hydrolyze the ester back to the initial organic acid. Accumulation of organic acids in the receiving phase was not observed in the absence of CRL due to their poor solubility in the ionic liquid used to construct the SLM. The use of ionic liquids as transport solvents also enabled long-term stability of the SLM compared to conventional organic solvent.

**Scheme 10:**
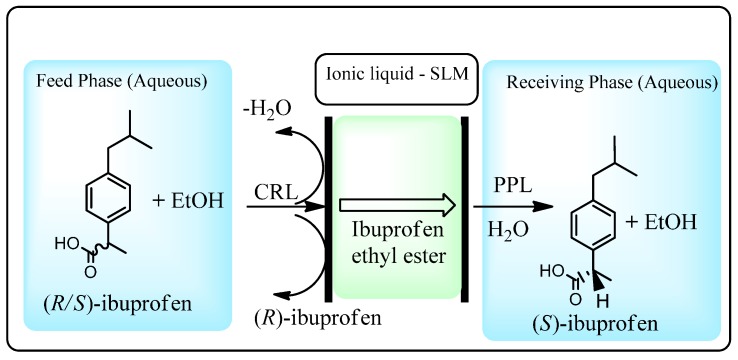
Lipase-facilitated enantioselective transport of (*S*)-ibuprofen across an ionic liquid based supported liquid membrane (SLM) [71].

This methodology was further extended to the resolution of (*S*)-ibuprofen through selective CRL-catalyzed esterification of the (*S*)-enantiomer in the feedstock, diffusion of the ester through the SLM ionic liquid and regeneration of (*S*)-ibuprofen in the receiving phase by PPL (Scheme 10) [71]. This process provided (*S*)-ibuprofen in up to 75% enantioselectivity.

**Scheme 11:**
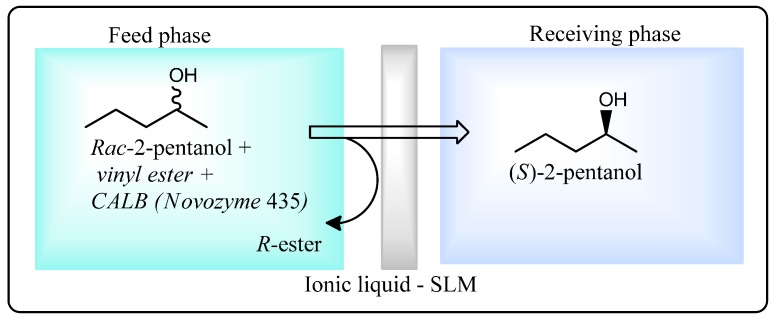
Lipase-facilitated resolution of *rac*-2-pentanol using an ionic liquid-based supported liquid membrane (SLM) [72].

An ionic liquid-based SLM system for the kinetic resolution of racemic 2-pentanol and separation of the unreacted enantiomer has been developed by Hernández-Fernández *et al.* [72]. (*S*)-2-Pentanol is an interesting chiral intermediate in the synthesis of drug candidates for Alzheimer’s disease [73]. Resolution and separation of (*S*)-2-pentanol from a racemic mixture was achieved by Novozym^®^ 435 lipase-catalyzed enantioselective transesterification of vinyl esters resulting in (*R*)-ester and (*S*)-alcohol (Scheme 11). In the SLM system, the alcohols readily diffused across the membrane whilst the diffusion rate of the esters was relatively low. Thus, enantioselective transesterification resulted in (*S*)-2-pentanol selectively accumulating in the receiving phase, enabling its separation from the (*R*)-enantiomer. Of six ionic liquids tested, [bmim][BF_4_] was found to be the most suitable as a transport solvent, giving 60–70-fold enrichment of the (*S*)-2-pentanol enantiomer in the receiving phase.

This methodology was further extended to the separation of (*S*)-phenylethanol, a commercially important chiral building block in the fine chemical and pharmaceutical industries [74]. The kinetic resolution of *rac*-1-phenylethanol was achieved by transesterification of a vinyl ester with the (*R*)-enantiomer, catalysed by Novozym^®^ 435 lipase. (*S*)-1-Phenylethanol and the (*R*)-vinyl ester derivative were separated in the reactor using a [bmim][BF_4_]-based SLM to give the (*S*)-enantiomer in 99% enantioselectivity.

#### 2.2.4. Biocatalysis in Biphasic Ionic Liquid-Supercritical Carbon Dioxide Systems

Some industrially important enzymes have been found to retain activity in a variety of non-aqueous solvents, including supercritical carbon dioxide (ScCO_2_) with several types of enzyme catalyzed reactions documented in this alternative solvent [75,76]. ScCO_2_ is an attractive industrial solvent owing to its non-flammable properties and low toxicity, and is relatively easily produced in terms of pressure and temperature compared to other supercritical fluids. A number of reports have demonstrated the value of combining ionic liquids and ScCO_2_ for green biocatalytic processes [42,77]. Because ScCO_2_ is immiscible with several different ionic liquids, it can be used to deliver substrates to, and extract products from, the ionic liquid phase. This particular use of ScCO_2_ makes it an attractive candidate for continuous flow and batch industrial processes for reactions involving the use of enzymes in ionic liquids. A critical feature of such systems is efficient mass transfer of substrates and products between ScCO_2_ and ionic liquids, which is dependent on ionic liquid hydrophobicity and viscosity.

**Scheme 12:**
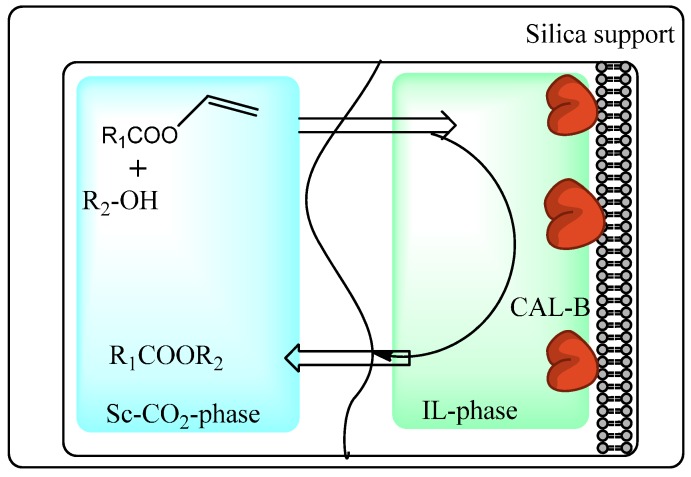
Transesterification of vinyl esters in a biphasic ionic liquid-ScCO_2_ system [78].

The group of Iborra have carried out detailed work on the use of biphasic systems consisting of ionic liquid and ScCO_2_ phases for combined biocatalysis and separation. Thus, Lozano *et al.* developed [78] a continuous process for CALB-catalyzed synthesis of short chain esters by transesterific-ation of vinyl esters with alcohols in an ionic liquid/Sc-CO_2_ biphasic system with ScCO_2_ acting as a substrate feedstock solvent and product extraction solvent (Scheme 12). Synthetic efficiency was greater in the biphasic system employing butyltetramethyl ammonium [btma][NTf_2_] compared to 3-cyanopropyl-trimethylammonium [NTf_2_] which was attributed to better mass transfer of substrates between ScCO_2_ and the more hydrophobic [btma][NTf_2_] ionic liquid.

**Figure 6:**
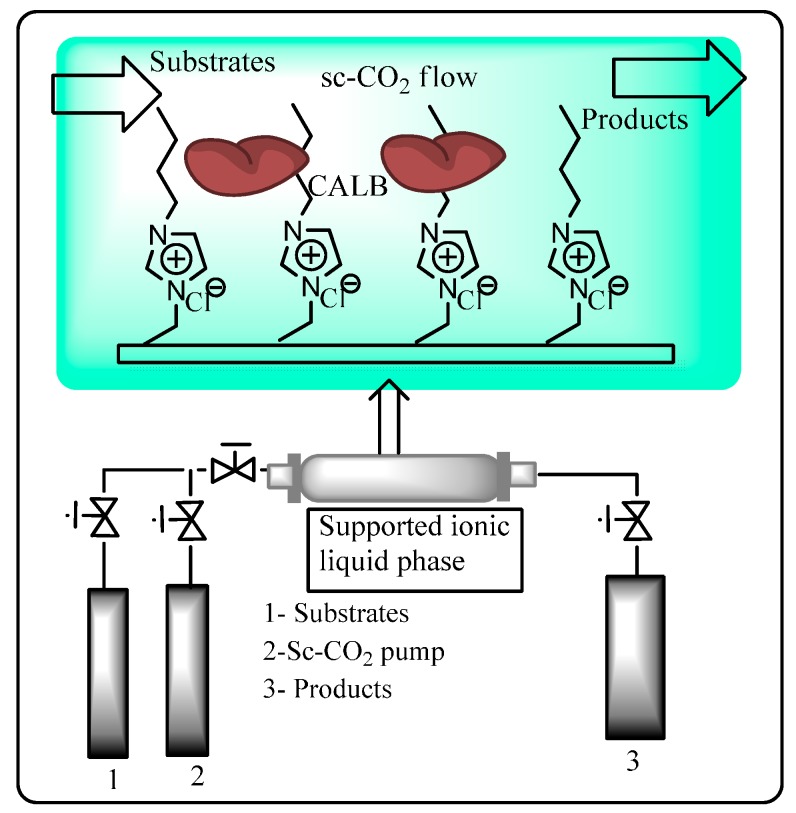
Continuous flow reactor setup employing CALB supported on an immobilized ionic liquid phase and sc-CO_2_ as a mobile phase [82].

Immobilization of ionic liquids onto an insoluble solid support is an attractive strategy for continuous biocatalytic processes employing a mobile ScCO_2_ phase since this approach minimizes the amount of ionic liquid used and enables easy product separation and catalyst recycling [79,80,81]. Lozano *et al* have synthesized immobilized ionic liquid phases by grafting or copolymerizing imidazolium-based ionic liquids to generate macroporous polymer monoliths with imidazolium loading ranging from 40% to 55% *w*/*w* [82]. The enzyme CALB was non-covalently adsorbed onto polymer monoliths from an aqueous solution. The systems were then used as heterogeneous catalysts for the continuous flow transesterification synthesis of citronellyl propionate from citronellol and vinyl propionate in ScCO_2_ (Figure 6). Productivity was highest when a styrene/divinylbenzene/imidazolium-based monolith was used compared to a 2-hydroxyethyl/ethylene glycol dimethyl acrylate/imidazolium-based monolith which was attributed to better mass transfer properties due to its greater hydrophobicity and porosity. Yields of up to 93% were achieved with no enzyme leaching observed.

The same group later reported studies on the immobilization of CALB by adsorption onto silica gel supports containing covalently grafted [btma] or trioctyl-methyl ammonium [toma][NTf_2_] ionic liquids [83]. The immobilized CALB was then assessed for its ability to kinetically resolve *rac*-1-phenylethanol by enantioselective transesterification of vinyl propionate in a continuous flow reactor using ScCO_2_ as the mobile phase. A yield of up to 96% and an enantioselectivity of >99% was achieved for the *trans*-esterification with the (*R*)-enantiomer. The productivity of this reaction system was found to be up to six times greater when using ScCO_2_ compared to hexane, attributed to better ability of ScCO_2_ to transport solutes through the grafted ionic liquid phase.

**Scheme 13:**
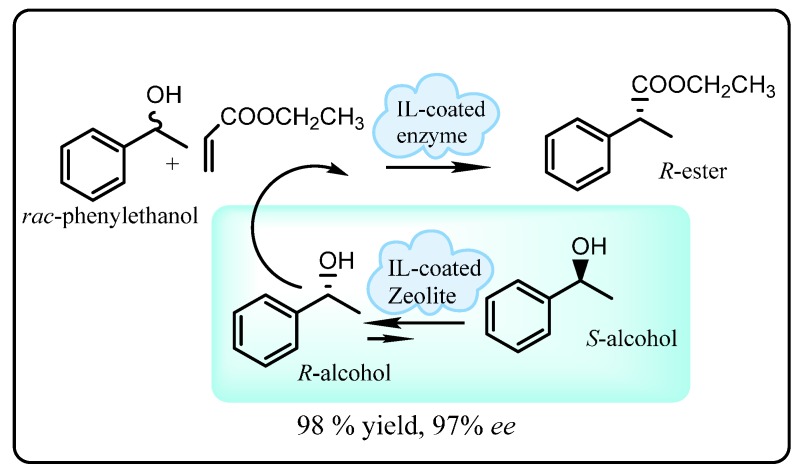
Dynamic kinetic resolution of *R*-phenylethylpropionate with ionic liquid-coated enzyme and zeolite [85].

In a seminal study, Lozano *et al.* [84,85] developed a continuous dynamic kinetic resolution of *rac*-1-phenylethanol employing a mixture of Novozym^®^ 435 lipase and a heterogeneous acidic zeolite in an ionic liquid-ScCO_2_ biphasic system. The role of the lipase was to catalyze transesterification of vinyl propionate with (*R*)-1-phenylethanol while the acidic zeolite was included to catalyze the isomerization of (*S*)-1-phenylethanol to the (*R*)-enantiomer. Both catalysts were first coated with ionic liquids by adsorption to stabilize the enzyme from the strongly acidic sites of the zeolite and the mobile ScCO_2_ phase. Overall, this catalytic system provided (*R*)-phenylethylpropionate in up to 98% yield and 97% enantioselectivity (Scheme 13) without any loss of catalyst activity after 14 days of operation. These findings clearly demonstrate the industrial potential of a combination of heterogeneous biological and chemical catalysis employing ionic liquids and ScCO_2_ in continuous flow process for green production of commercially important enantiomers.

#### 2.2.5. Use of Biodegradable Ionic-Based Deep Eutectic Solvents for Biocatalysis

Although ionic liquids are often claimed to be new generation green solvents due to their low flammability and vapor pressure, their component ions may be toxic, slow to biodegrade and may accumulate in marine organisms. Hence, the presence of ionic liquids in industrial waste streams may result in an environmental hazard. In light of this potential problem, the design and use of biodegradable ionic liquids that will not persist in the environment is a matter of importance. The building blocks used to synthesize such a class of ionic liquids are generally those regarded as being biocompatible, e.g., amines, amino acids, amino alcohols, essential oils, carbohydrates and carboxylic acids [86]. An added advantage is that these building blocks are obtained from renewable resources, which further enhances the sustainability profile of the ionic liquid.

An interesting class of designed biodegradable ionic liquids are physical mixtures of biological salts, such as choline chloride and uncharged biological hydrogen bond donors, e.g., urea or glycerol, which exist as deep eutectic solvents (DESs). Such eutectic solvents exist as a physical mixture with a specific component composition that imparts a melting temperature much lower than that of any of the individual components. Compared to earlier generation ionic liquids, ionic-based DESs are relatively easy to prepare in accord with green chemistry principles since they simply comprised of mixtures of compounds in defined ratios and hence do not require any purification [43].

Currently, there are only a few examples of enzyme catalysis in a DES compared to earlier generations of ionic liquids. The first report of enzymes used economically with a DES was a lipase-catalyzed transesterification of ethyl valerate with 1-butanol [87]. The specific activity of immobilized CALB (iCALB) was found to be 2–7 times higher in a choline chloride:glycerol (ChCl:Gly) DES compared to earlier generation ionic liquids [bmim][Tf_2_N] or [bmim][BF_4_], with conversion of 96% achieved. Remarkably, transesterification with the glycerol component of the DES was extremely low (<0.5%). iCALB-catalysed aminolysis of ethyl valerate with 1-butylamine also proceeded efficiently in ChCl:Gly with >90% conversion after 1 h. ChCl:Gly was also found to be a suitable co-solvent with aqueous buffers; a significant 20-fold enhancement in the rate of epoxide hydrolase-catalyzed hydrolysis of styrene oxide was observed in 25% *v*/*v* ChCl:Gly/buffer compared to buffer alone.

**Scheme 14:**
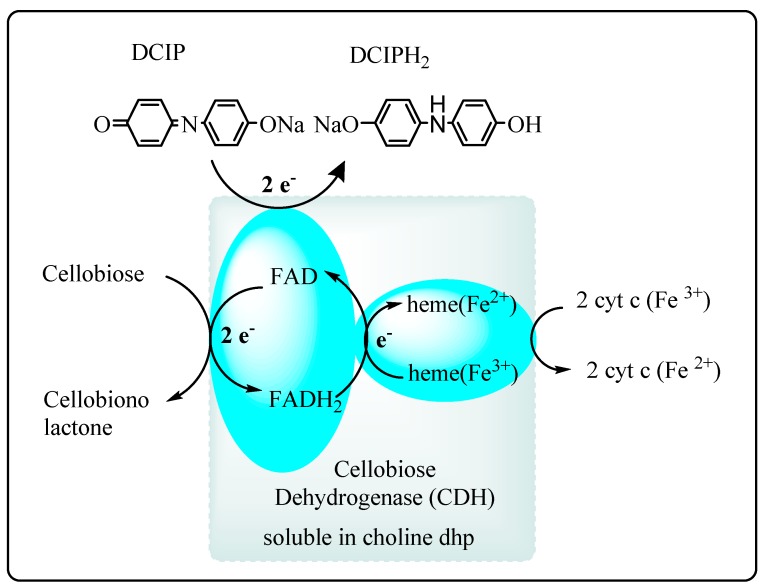
CDH-catalyzed oxidation of cellobiose by inter and intra-electron transfer pathways [88].

The biocatalytic oxidation of cellulose-derived carbohydrates has potential application in bioelectrochemical synthesis and energy production in biofuel cells. Recently, the potential of a biocompatible hydrated choline dihydrogen phosphate (choline dhp) ionic liquid containing 35 wt % water as a solvent for the cellobiose dehydrogenase (CDH)-catalyzed oxidation of cellobiose to cellobionolactone (Scheme 14) was demonstrated [88]. Inter-electron transfer to a synthetic dye acceptor, DCIP, was observed, indicating the system may also have use in bioelectrochemical synthesis. Since CDH is known to be able to transfer electrons to electrodes, the CDH-DES system may have application in ionic liquid/carbohydrate-based bioenergy cells.

Recently, Zhao *et al* have employed DES-made from choline acetate and glycerol in the enzymatic preparation of biodiesel from Miglyol^®^ oil 812 [89]. A eutectic mixture of choline acetate/glycerol (1:1.5) had a lower viscosity than choline chloride/urea eutectic mixtures, and was found to support CALB enzyme activity. Transesterification reactions performed in DES and 20% (*v*/*v*) MeOH and 1% water using Novozym^®^ 435 gave a faster reaction rate compared to that in organic solvents or hydrophobic ionic liquids, and afforded 97% conversion. The biphasic separation of the resultant biodiesel allowed for its easy separation from the DES.

### 2.3. Application of Ionic Liquids in Whole-Cell Biocatalysis: A Greener Perspective

Biocatalysis in organic synthesis employing recombinant enzymes requires an expensive set of enzyme purification unit operations, which themselves generate undesirable waste streams. Using whole cells to catalyze chemical transformations avoids this problem. However, technological challenges with whole-cell biocatalysis often include poor substrate and product solubility in the aqueous buffer/medium critical for cell survival. Furthermore, product accumulation in the cell culture medium can lead to cell toxicity. To overcome these problems, multiphase solvent systems have been developed using immiscible organic solvents, which act as substrate reservoirs and product extraction agents. However, the limitations of these systems are the flammability/toxicity of these solvents, and their deleterious effects on cell viability due to their destructive effects on cell membrane structure [77]. Hydrophobic ionic liquids are an attractive alternative to overcome these problems. Compared to conventional organic solvents, the ionic liquids [bmim][PF_6_], [bmim][NTF_2_] and methytrioctyl-ammonium [NTf_2_] have been found not to affect cell membrane integrity of *Lactobacillus kefir*, *Escherichia coli* and *Saccharomyces cerevisiae* in biphasic systems, thus offering the advantage of preserving cell function and allowing intracellular co-factor regeneration in organic synthesis.

**Scheme 15:**
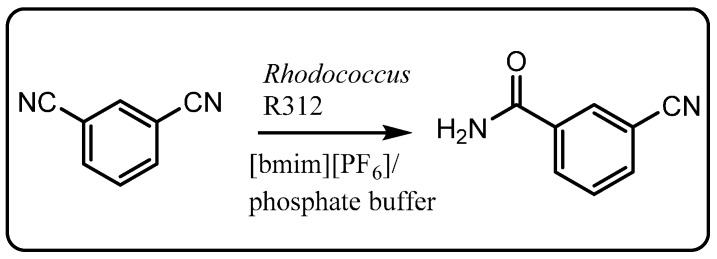
Hydration of 1,3-dicyanobenzene to 3-cyanobenzamide using *Rhodococcus* R312 in biphasic system [90].

The first example of whole-cell biocatalysis employing a water-ionic liquid biphasic system was reported by Cull *et al.* using *Rhodococcus* R312 [90]. These cells contain nitrile hydratase which catalyzes the hydration of the nitrile group to an amide, a process of synthetic interest for producing fine chemicals and pharmaceutical intermediates. Toluene could be replaced by [bmim][PF_6_] as a reservoir for the substrate 1,3-dicyanobenzene and as an extraction solvent for the product 3-cyano-benzamide in this biphasic system (Scheme 15). It was observed that the initial rate of 3-cyanobenz-amide production was lower in the [bmim][PF_6_]-water biphasic system compared to the toluene-water system but that the overall yield was slightly higher. Since the specific activity of nitrile hydratase activity was found to be higher in the ionic liquid-water system and that the ionic liquid did not reduce cell viability, it was concluded the lower rate of product formation was due to reduced mass transfer of substrate from the more viscous ionic liquid phase. An advantage of [bmim][PF_6_] in downstream processing was reduced cell aggregation at the phase interface, which made product recovery and material recycling easier.

**Scheme 16:**
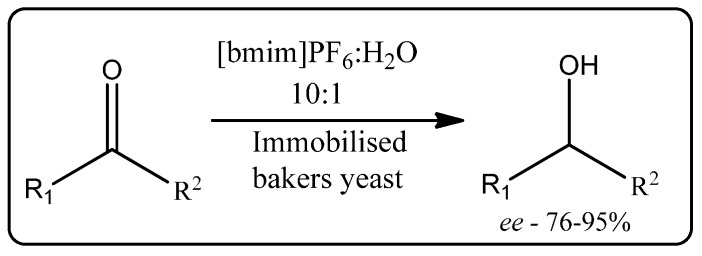
Immobilized bakers yeast-catalyzed reduction of ketones in a biphasic ionic liquid-water system [91].

Asymmetric reduction of prochiral ketones to chiral alcohols is an important reaction in the development of chiral pharmaceuticals. Howarth *et al.* have reported whole-cell biocatalysis for asymmetric reduction of prochiral ketones in a biphasic ionic liquid-water system [91]. In this work, yeast was immobilized by encapsulation in low-cost calcium alginate beads and the reduction of several different prochiral ketones performed in a biphasic system of water and [bmim][PF_6_] containing the substrate (Scheme 16). Yields ranged from poor to good depending on the chosen substrate, with some excellent enantioselectivities (>95%) obtained. The asymmetric reduction of prochiral ketones in ionic liquid-water biphasic systems has since been further developed [92,93].

**Scheme 17:**
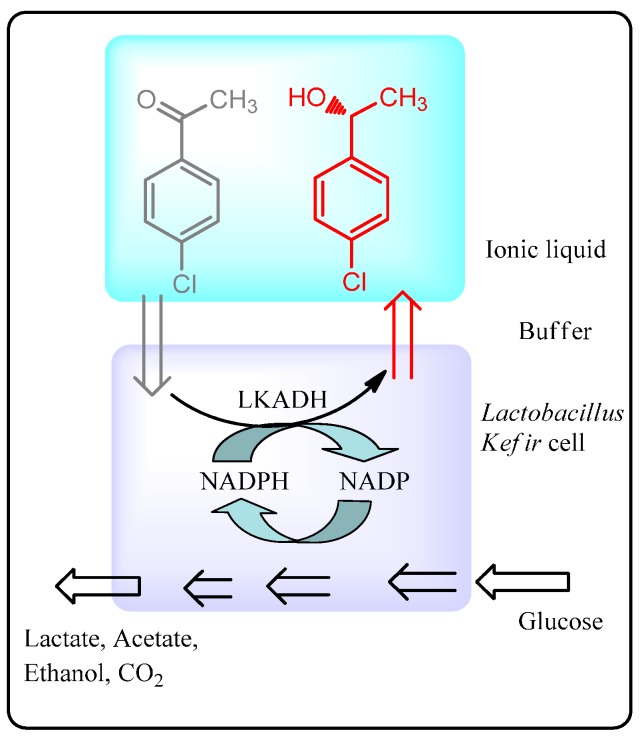
Asymmetric reduction of 4-chloroacetophenone by *Lactobacillus kefir* in a biphasic ionic liquid-water system [92].

Asymmetric reduction of 4-chloro-acetophenone to (*R*)-1-(4-chlorophenyl)ethanol with *Lactobacillus* *kefir* was carried out as a representative example of a whole-cell-catalyzed multiphase process for the synthesis of fine chemicals (Scheme 17) [92]. In this biphasic system, the ionic liquids employed did not disrupt the necessary cellular cofactor generation systems required for performing the reaction, thus eliminating the need to add these expensive cofactors externally, and provided the product in 92.8% yield with 99.7% enantioselectivity. The potential industrial application of this system was demonstrated by scaling up the reaction volume nearly a hundred fold with the same yield and product purity. Furthermore, no emulsion formed when mixing the two phases, enabling their easy separation for subsequent product recovery and solvent recycling. Similar biphasic ionic liquid-water systems have been used for the *Saccharomyces cerevisiae* asymmetric reduction of 4-chloro acetoacetate to (*S*)-4-chloro-3-hydroxy-butanoate, an intermediate in the synthesis of cholesterol-reducing statin inhibitors, in good yield (80%) and enantioselectivity (84%). In another example of asymmetric reduction, Xu *et al.* [93] used immobilized *Acetobacter* sp. CCTCC M209061 cells in a deep eutectic comprised of chloline chloride and urea as a biocatalytic system to reduce 3-chloro-propiophenone to (*S*)-3chloro-1-phenylpropanol in 82% yield and >99% enantioselectivity. The reaction could be scaled up to 500 mL with comparable yield and enantioselectivity.

In addition to being employed in whole-cell biphasic systems for the production of fine chemicals or pharmaceutical intermediates, ionic liquids have also been assessed as post-fermentation extraction solvents of amino acids and antibiotics [90,93,94,95,96]. The partition coefficients of erythromycin in biphasic water-butyl acetate and water-[bmim][PF_6_] were, for example, found by Pfruender *et al.* to be comparable at physiological pH, with about a 10-fold accumulation into the non-aqueous phase [93]. At pH 10 or above, partitioning of erythromycin into [bmim][PF_6_] decreased to 2-fold while accumulation into butyl acetate increased. These results suggest a potential use of [bmim][PF_6_] as a non-flammable extraction solvent for erythromycin at physiological pH followed by back extraction with an alkaline aqueous solution to enable recycling of the ionic liquid. Similarly, a pH-dependent partitioning of the antibiotics amoxicillin and ampicllin between water and ionic liquids was observed by Soto *et al.*; the antibiotics accumulated 3–5-fold into ionic liquid at pH 8 while the reverse occurred at pH 4 [96]. Many different amino acids are prepared by cell fermentation but their isolation by liquid-liquid extraction technologies can be problematic due to their hydrophilic properties. Smirnova *et al.* assessed the ability of a solution of the crown ether dicyclohexano-18-crown-6 in [bmim][PF_6_] to extract amino acids from aqueous solution [95]. In this system, the crown ether served as a complexing agent with the ammonium group at acidic pH to enhance the solubility of the amino acid in the ionic liquid. Excellent recoveries (>90%) of hydrophilic and hydrophobic amino acids were obtained from acidic aqueous solutions and fermentation broths. Furthermore, the amino acids were efficiently back-extracted from the ionic-liquid using an alkaline aqueous solution.

Although enzyme-based production of biodiesel is an attractive alternative to homogenous alkali catalysis, a whole-cell process for producing biodiesel would remove the need for an enzyme purification step. To this end, Arai *et al.* demonstrated the transesterification of soybean oil with methanol using fungi in an ionic liquid-oil biphasic system with [emim][BF_4_] and [bmim][BF_4_] [97]. These ionic liquids were chosen to act as a reservoir for methanol and the transesterification by-product glycerol, both of which can have detrimental effects on whole cell catalysts, while allowing phase separation of the biodiesel for easy product separation. Four types of whole-cell biocatalysts supported on polyurethane biomass support particles were assessed in this study; wild type *Rhizopus oryzae* producing triacylglycerol lipase (w-ROL) and genetically engineered *Aspergillus oryzae* expressing either *Fusarium heterosporum* lipase (r-FHL), *Candida antarctica* lipase B (r-CALB) or *A. oryzae* mono- and diacylglycerol lipase (r-mdlB). Supported w-ROL was found to have the highest activity achieving 60% FAME content. Its use alone did not result in complete FAME production because of the inherent positional specificity of its triacylglycerol lipase. However, a higher FAME content of 90% was achieved using a mixture of supported w-ROL and r-mdlB. A difficulty associated with the long-term stability of w-ROL in the presence of ionic liquid was overcome by treating it with glutaraldehyde to cross-link its endogenous lipases. This greatly stabilized w-ROL and enabled its recycling. Overall, this research demonstrated the potential application of ionic liquids and whole-cell biocatalysis in developing a more sustainable and environmentally friendly route toward biodiesel production.

## 3. Conclusions

Ionic liquids as bulk solvent phases offer new opportunities as recyclable alternatives to conventional organic solvents for biocatalytic production of commercially important chemicals including asymmetric synthesis, with enhancement in certain cases of the rate of product formation and enantioselectivity. The application of benign ionic liquids that can both dissolve cellulose and maintain enzyme activity bodes well for future developments in utilizing cellulose as raw chemical resource. Similar strategies can be forecasted as apparent from our recent work with biphasic biocatalytic conversion of xylan, the second most abundant biomaterial found in nature after cellulose, using recombinantly expressed, thermostable xylanases, genetically engineered to have high activity in a variety of non-polar ionic liquids, leading to the production of a range of chemical intermediates that can be directly recovered from the aqueous phase in continuous processes [98]. Similar approaches and processes are equally germane in applications with biphasic ionic liquid-vegetable oil mixtures and show great potential as efficient reaction systems for producing biodiesel using enzyme or whole-cell-catalyzed transesterification. In addition to their use as bulk solvents, ionic liquids have been applied as enzyme coatings for the development of recyclable heterogeneous biocatalysts, as immobilized supports for enzymes in biphasic systems employing ScCO_2_ for continuous reaction systems and as components of supported liquid membranes for continuous separation of reactants and products in enzyme catalyzed reactions. These are exciting developments as the amounts of ionic liquid required in these systems are much less than those for bulk solvent applications, which will reduce any unfavorable environmental impacts of synthesizing or using ionic liquids. The inherent biphasic nature of these systems also enables easier recycling of the ionic liquid component. The CDH-catalyzed oxidation of cellobiose in a biodegradable ionic-based DES demonstrates the potential of employing ionic liquids as components of bioelectrochemical energy cells. Overall, these developments highlight the potential of ionic liquid-based biocatalytic processes in designing more benign sustainable chemical processes for enhancing the quality of life of future generations.

## References

[1] Anastas P.T., Kirchhoff M.M. (2002). Origins, current status, and future challenges of green chemistry. Acc. Chem. Res..

[2] Welton T. (1999). Room-temperature ionic liquids. Solvents for synthesis and catalysis. Chem. Rev..

[3] MacFarlane D.R., Forsyth M., Howlett P.C., Pringle J.M., Sun J., Annat G., Neil W., Izgorodina E.I. (2007). Ionic liquids in electrochemical devices and processes: Managing interfacial electrochemistry. Acc. Chem. Res..

[4] Dupont J., de Souza R.F., Suarez P.A.Z. (2002). Ionic liquid (molten salt) phase organometallic catalysis. Chem. Rev..

[5] Roosen C., Muller P., Greiner L. (2008). Ionic liquids in biotechnology: Applications and perspectives for biotransformations. Appl. Microbiol. Biotechnol..

[6] Wang Z.S., Koumura N., Cui Y., Miyashita M., Mori S., Hara K. (2009). Exploitation of ionic liquid electrolyte for dye-sensitized solar cells by molecular modification of organic-dye sensitizers. Chem. Mater..

[7] Riisager A., Fehrmann R., Haumann M., Wasserscheid P. (2006). Supported ionic liquid phase (silp) catalysis: An innovative concept for homogeneous catalysis in continuous fixed-bed reactors. Eur. J. Inorg. Chem..

[8] Gruttadauria M., Riela S., Aprile C., Lo Meo P., D’Anna F., Noto R. (2006). Supported ionic liquids. New recyclable materials for the l-proline-catalyzed aldol reaction. Adv. Synth. Catal..

[9] Wasserscheid P., Keim W. (2000). Ionic liquids—New “solutions” for transition metal catalysis. Angew. Chem. Int. Ed..

[10] Fei Z.F., Geldbach T.J., Zhao D.B., Dyson P.J. (2006). From dysfunction to bis-function: On the design and applications of functionalised ionic liquids. Chem. -Eur. J..

[11] Lee S.G. (2006). Functionalized imidazolium salts for task-specific ionic liquids and their applications. Chem. Commun..

[12] De Maria P.D. (2008). “Nonsolvent” applications of ionic liquids in biotransformations and organocatalysis. Angew. Chem. Int. Ed..

[13] Van Rantwijk F., Sheldon R.A. (2007). Biocatalysis in ionic liquids. Chem. Rev..

[14] Miao W.S., Chan T.H. (2005). Ionic-liquid-supported peptide synthesis demonstrated by the synthesis of leu^5^-enkephalin. J. Org. Chem..

[15] Fraga-Dubreuil J., Bazureau J.P. (2001). Grafted ionic liquid-phase-supported synthesis of small organic molecules. Tetrahedron Lett..

[16] Deetlefs M., Seddon K.R. (2010). Assessing the greenness of some typical laboratory ionic liquid preparations. Green Chem..

[17] Harjani J.R., Singer R.D., Garciac M.T., Scammells P.J. (2009). Biodegradable pyridinium ionic liquids: Design, synthesis and evaluation. Green Chem..

[18] Harjani J.R., Farrell J., Garcia M.T., Singer R.D., Scammells P.J. (2009). Further investigation of the biodegradability of imidazolium ionic liquids. Green Chem..

[19] Tao J.H., Zhao L.S., Ran N.Q. (2007). Recent advances in developing chemoenzymatic processes for active pharmaceutical ingredients. Org. Process Res. Dev..

[20] Straathof A.J.J., Panke S., Schmid A. (2002). The production of fine chemicals by biotransformations. Curr. Opin. Biotechnol..

[21] Klibanov A.M. (2001). Improving enzymes by using them in organic solvents. Nature.

[22] Klibanov A.M. (1989). Enzymatic catalysis in anhydrous organic-solvents. Trends Biochem. Sci..

[23] Magnuson D.K., Bodley J.W., Evans D.F. (1984). The activity and stability of alkaline phosphatase in solutions of water and the fused salt ethylammonium nitrate. J. Sol. Chem..

[24] Moniruzzaman M., Nakashima K., Kamiya N., Goto M. (2010). Recent advances of enzymatic reactions in ionic liquids. Biochem. Eng. J..

[25] Zhao H. (2010). Methods for stabilizing and activating enzymes in ionic liquids. J. Chem. Technol. Biotechnol..

[26] Moniruzzaman M., Kamiya N., Goto M. (2010). Activation and stabilization of enzymes in ionic liquids. Org. Biomol. Chem..

[27] Yang Z. (2009). Hofmeister effects: An explanation for the impact of ionic liquids on biocatalysis. J. Biotechnol..

[28] Zhao H. (2005). Effect of ions and other compatible solutes on enzyme activity, and its implication for biocatalysis using ionic liquids. J. Mol. Catal. B Enzym..

[29] De Gonzalo G., Lavandera I., Durchschein K., Wurm D., Faber K., Kroutil W. (2007). Asymmetric biocatalytic reduction of ketones using hydroxy-functionalised water-miscible ionic liquids as solvents. Tetrahedron Asymmetry.

[30] Lau R.M., Sorgedrager M.J., Carrea G., van Rantwijk F., Secundo F., Sheldon R.A. (2004). Dissolution of candida antarctica lipase b in ionic liquids: Effects on structure and activity. Green Chem..

[31] Erbeldinger M., Mesiano A.J., Russell A.J. (2000). Enzymatic catalysis of formation of z-aspartame in ionic liquid—An alternative to enzymatic catalysis in organic solvents. Biotechnol. Prog..

[32] Lau R.M., van Rantwijk F., Seddon K.R., Sheldon R.A. (2000). Lipase-catalyzed reactions in ionic liquids. Org. Lett..

[33] Harjani J.R., Naik P.U., Nara S.J., Salunkhe M.M. (2007). Enzyme mediated reactions in ionic liquids. Curr. Org. Synth..

[34] Yang Z., Pan W.B. (2005). Ionic liquids: Green solvents for nonaqueous biocatalysis. Enzym. Microb. Technol..

[35] Sureshkumar M., Lee C.K. (2009). Biocatalytic reactions in hydrophobic ionic liquids. J. Mol. Catal. B Enzym..

[36] Sheldon R.A. (2014). Biocatalysis in ionic liquids. RSC Catal. Ser..

[37] Lozano P., Bernal J.M., Garcia-Verdugo E., Vaultier M., Luis S.V. (2015). Biocatalysis in Ionic Liquids.

[38] Stein F., Kragl U. (2014). Biocatalytic Reactions in Ionic Liquids.

[39] Klembt S., Dreyer S., Eckstein M., Kragl U., Wasserscheid P., Welton T. (2008). Biocatalytic reactions in ionic liquids. Ionic Liquids in Synthesis.

[40] Schöfer S.H., Kaftzik N., Wasserscheid P., Kragl U. (2001). Enzyme catalysis in ionic liquids: Lipase catalysed kinetic resolution of 1-phenylethanol with improved enantioselectivity. Chem. Commun..

[41] Itoh T., Akasaki E., Kudo K., Shirakami S. (2001). Lipase-catalyzed enantioselective acylation in the ionic liquid solvent system: Reaction of enzyme anchored to the solvent. Chem. Lett..

[42] Cantone S., Hanefeld U., Basso A. (2007). Biocatalysis in non-conventional media-ionic liquids, supercritical fluids and the gas. Green Chem..

[43] Gorke J., Srienc F., Kazlauskas R. (2010). Toward advanced ionic liquids. Polar, enzyme-friendly solvents for biocatalysis. Biotechnol. Bioprocess Eng..

[44] Mohile S.S., Potdar M.K., Harjani J.R., Nara S.J., Salunkhe M.M. (2004). Ionic liquids: Efficient additives for candida rugosa lipase-catalyzed enantioselective hydrolysis of butyl 2-(4-chlorophenoxy)propionate. J. Mol. Catal. B Enzym..

[45] Rasalkar M.S., Potdar M.K., Salunkhe M.M. (2004). Pseudomonas cepacia lipase-catalysed resolution of racemic alcohols in ionic liquid using succinic anhydride: Role of triethylamine in enhancement of catalytic activity. J. Mol. Catal. B Enzym..

[46] Lourenco N.M.T., Barreiros S., Afonso C.A.M. (2007). Enzymatic resolution of indinavir precursor in ionic liquids with reuse of biocatalyst and media by product sublimation. Green Chem..

[47] Mai N.L., Ahn K., Bae S.W., Shin D.W., Morya V.K., Koo Y.-M. (2014). Ionic liquids as novel solvents for the synthesis of sugar fatty acid ester. Biotechnol. J..

[48] Lee J.K., Kim M.-J. (2002). Ionic liquid-coated enzyme for biocatalysis in organic solvent. J. Org. Chem..

[49] Itoh T., Matsushita Y., Abe Y., Han S.-H., Wada S., Hayase S., Kawatsura M., Takai S., Morimoto M., Hirose Y. (2006). Increased enantioselectivity and remarkable acceleration of lipase-catalyzed transesterification by using an imidazolium peg-alkyl sulfate ionic liquid. Chem. Eur. J..

[50] Nakashima K., Kamiya N., Koda D., Maruyama T., Goto M. (2009). Enzyme encapsulation in microparticles composed of polymerized ionic liquids for highly active and reusable biocatalysts. Org. Biomol. Chem..

[51] Yu C.-Y., Wei P., Li X.-F., Zong M.-H., Lou W.-Y. (2014). Using ionic liquid in a biphasic system to improve asymmetric hydrolysis of styrene oxide catalyzed by cross-linked enzyme aggregates (cleas) of mung bean epoxide hydrolases. Ind. Eng. Chem. Res..

[52] Rehmann L., Ivanova E., Gunaratne H.Q.N., Seddon K.R., Stephens G. (2014). Enhanced laccase stability through mediator partitioning into hydrophobic ionic liquids. Green Chem..

[53] Turner M.B., Spear S.K., Huddleston J.G., Holbrey J.D., Rogers R.D. (2003). Ionic liquid salt-induced inactivation and unfolding of cellulase from trichoderma reesei. Green Chem..

[54] Toral A.R., de los Rios A.P., Hernandez F.J., Janssen M.H.A., Schoevaart R., van Rantwijk F., Sheldon R.A. (2007). Cross-linked candida antarctica lipase b is active in denaturing ionic liquids. Enzym. Microb. Technol..

[55] Zhao H., Baker G.A., Song Z., Olubajo O., Crittle T., Peters D. (2008). Designing enzyme-compatible ionic liquids that can dissolve carbohydrates. Green Chem..

[56] Vancov T., Alston A.-S., Brown T., McIntosh S. (2012). Use of ionic liquids in converting lignocellulosic material to biofuels. Renew. Energy.

[57] Long J., Li X., Guo B., Wang F., Yu Y., Wang L. (2012). Simultaneous delignification and selective catalytic transformation of agricultural lignocellulose in cooperative ionic liquid pairs. Green Chem..

[58] Shi J., Gladden J.M., Sathitsuksanoh N., Kambam P., Sandoval L., Mitra D., Zhang S., George A., Singer S.W., Simmons B.A. (2013). One-pot ionic liquid pretreatment and saccharification of switchgrass. Green Chem..

[59] Sun N., Parthasarathi R., Socha A.M., Shi J., Zhang S., Stavila V., Sale K.L., Simmons B.A., Singh S. (2014). Understanding pretreatment efficacy of four cholinium and imidazolium ionic liquids by chemistry and computation. Green Chem..

[60] Moniruzzaman M., Ono T. (2012). Ionic liquid assisted enzymatic delignification of wood biomass: A new green’ and efficient approach for isolating of cellulose fibers. Biochem. Eng. J..

[61] Melero J.A., Iglesias J., Morales G. (2009). Heterogeneous acid catalysts for biodiesel production: Current status and future challenges. Green Chem..

[62] Ha S.H., Lan M.N., Lee S.H., Hwang S.M., Koo Y.-M. (2007). Lipase-catalyzed biodiesel production from soybean oil in ionic liquids. Enzym. Microb. Technol..

[63] Gamba M., Lapis A.A.M., Dupont J. (2008). Supported ionic liquid enzymatic catalysis for the production of biodiesel. Adv. Synth. Catal..

[64] Zhao H., Song Z., Olubajo O., Cowins Janet V. (2010). New ether-functionalized ionic liquids for lipase-catalyzed synthesis of biodiesel. Appl. Biochem. Biotechnol..

[65] Yu D., Wang C., Yin Y., Zhang A., Gao G., Fang X. (2011). A synergistic effect of microwave irradiation and ionic liquids on enzyme-catalyzed biodiesel production. Green Chem..

[66] Zhang K.P., Lai J.Q., Huang Z.L., Yang Z. (2011). Penicillium expansum lipase-catalyzed production of biodiesel in ionic liquids. Bioresour. Technol..

[67] Lai J.-Q., Hu Z.-L., Wang P.-W., Yang Z. (2012). Enzymatic production of microalgal biodiesel in ionic liquid [bmim][pf6]. Fuel.

[68] Pellegrino J.J., Noble R.D. (1990). Enhanced transport and liquid membranes in bioseparations. Trends Biotechnol..

[69] Branco L.C., Crespo J.G., Afonso C.A.M. (2002). Highly selective transport of organic compounds by using supported liquid membranes based on ionic liquids. Angew. Chem. Int. Ed..

[70] Miyako E., Maruyama T., Kamiya N., Goto M. (2003). Use of ionic liquids in a lipase-facilitated supported liquid membrane. Biotechnol. Lett..

[71] Miyako E., Maruyama T., Kamiya N., Goto M. (2003). Enzyme-facilitated enantioselective transport of (*S*)-ibuprofen through a supported liquid membrane based on ionic liquids. Chem. Commun..

[72] Hernandez-Fernandez F.J., de los Rios A.P., Tomas-Alonso F., Gomez D., Villora G. (2008). On the development of an integrated membrane process with ionic liquids for the kinetic resolution of rac-2-pentanol. J. Membr. Sci..

[73] Audia J.E., Britton T.C., Droste J.J., Folmer B.K., Huffman G.W., John V., Latimer L.H., Mabry T.E., Nissen J.S. (1998). Preparation of *N*-(phenylacetyl)di- and Tripeptide Derivatives for Inhibiting β-Amyloid Peptide Release.

[74] Hernandez-Fernandez F.J., de los Rios A.P., Tomas-Alonso F., Gomez D., Villora G. (2009). Kinetic resolution of 1-phenylethanol integrated with separation of substrates and products by a supported ionic liquid membrane. J. Chem. Technol. Biotechnol..

[75] Kamat S.V., Beckman E.J., Russell A.J. (1995). Enzyme-activity in supercritical fluids. Crit. Rev. Biotechnol..

[76] Mesiano A.J., Beckman E.J., Russell A.J. (1999). Supercritical biocatalysis. Chem. Rev..

[77] Lozano P. (2010). Enzymes in neoteric solvents: From one-phase to multiphase systems. Green Chem..

[78] Lozano P., de Diego T., Gmouh S., Vaultier M., Iborra J.L. (2004). Criteria to design green enzymatic processes in ionic liquid/supercritical carbon dioxide systems. Biotechnol. Prog..

[79] Fonseca G.S., Scholten J.D., Dupont J. (2004). Iridium nanoparticles prepared in ionic liquids: An efficient catalytic system for the hydrogenation of ketones. Synlett.

[80] Valkenberg M.H., de Castro C., Holderich W.F. (2002). Immobilisation of ionic liquids on solid supports. Green Chem..

[81] Mehnert C.P. (2004). Supported ionic liquid phases. Chem. -Eur. J..

[82] Lozano P., Garcia-Verdugo E., Piamtongkam R., Karbass N., De Diego T., Burguete M.I., Luis S.V., Iborra J.L. (2007). Bioreactors based on monolith-supported ionic liquid phase for enzyme catalysis in supercritical carbon dioxide. Adv. Synth. Catal..

[83] Lozano P., De Diego T., Sauer T., Vaultier M., Gmouh S., Iborra J.L. (2007). On the importance of the supporting material for activity of immobilized candida antarctica lipase b in ionic liquid/hexane and ionic liquid/supercritical carbon dioxide biphasic media. J. Supercrit. Fluids.

[84] Lozano P., de Diego T., Mira C., Montague K., Vaultier M., Iborra J.L. (2009). Long term continuous chemoenzymatic dynamic kinetic resolution of rac-1-phenylethanol using ionic liquids and supercritical carbon dioxide. Green Chem..

[85] Lozano P., de Diego T., Vaultier M., Iborra J.L. (2009). Dynamic kinetic resolution of sec-alcohols in ionic liquids/supercritical carbon dioxide biphasic systems. Int. J. Chem. React. Eng..

[86] Imperato G., Konig B., Chiappe C. (2007). Ionic green solvents from renewable resources. Eur. J. Org. Chem..

[87] Gorke J.T., Srienc F., Kazlauskas R.J. (2008). Hydrolase-catalyzed biotransformations in deep eutectic solvents. Chem. Commun..

[88] Fujita K., Nakamura N., Igarashi K., Samejima M., Ohno H. (2009). Biocatalytic oxidation of cellobiose in an hydrated ionic liquid. Green Chem..

[89] Zhao H., Baker G.A., Holmes S. (2011). New eutectic ionic liquids for lipase activation and enzymatic preparation of biodiesel. Org. Biomol. Chem..

[90] Cull S.G., Holbrey J.D., Vargas-Mora V., Seddon K.R., Lye G.J. (2000). Room-temperature ionic liquids as replacements for organic solvents in multiphase bioprocess operations. Biotechnol. Bioeng..

[91] Howarth J., James P., Dai J. (2001). Immobilized baker’s yeast reduction of ketones in an ionic liquid, [bmim]PF6 and water mix. Tetrahedron Lett..

[92] Pfruender H., Midjojo M., Kragl U., Weuster-Botz D. (2004). Efficient whole-cell biotransformation in a biphasic ionic liquid/water system. Angew. Chem. Int. Ed..

[93] Xu P., Xu Y., Li X.-F., Yi Zhao B.-Y., Zong M.-H., Lou W.-Y. (2015). Enhancing Asymmetric Reduction of 3-Chloropropiophenone with Immobilized Acetobacter sp. CCTCC M209061 Cells by Using Deep Eutectic Solvents as Cosolvents. ACS Sustain. Chem. Eng..

[94] Pfruender H., Jones R., Weuster-Botz D. (2006). Water immiscible ionic liquids as solvents for whole cell biocatalysis. J. Biotechnol..

[95] Smirnova S.V., Torocheshnikova I.I., Formanovsky A.A., Pletnev I.V. (2004). Solvent extraction of amino acids into a room temperature ionic liquid with dicyclohexano-18-crown-6. Anal. Bioanal. Chem..

[96] Soto A., Arce A., Khoshkbarchi M.K. (2005). Partitioning of antibiotics in a two-liquid phase system formed by water and a room temperature ionic liquid. Sep. Purif. Technol..

[97] Arai S., Nakashima K., Tanino T., Ogino C., Kondo A., Fukuda H. (2010). Production of biodiesel fuel from soybean oil catalyzed by fungus whole-cell biocatalysts in ionic liquids. Enzym. Microb. Technol..

[98] Lim A., Zhang C., Oktavianawati I., Hearn M.T.W. (2015). Continuous enzymatic conversion of xylan with product recovery by liquid-liquid two-phase extraction.

